# The analysis of waiting time and utilization of computed tomography and magnetic resonance imaging in Croatia: a nationwide survey

**DOI:** 10.3325/cmj.2020.61.538

**Published:** 2020-12

**Authors:** Zrinka Biloglav, Petar Medaković, Jure Buljević, Franko Žuvela, Ivan Padjen, Dina Vrkić, Josip Ćurić

**Affiliations:** 1 Department of Medical Statistics, Epidemiology and Medical Informatics, School of Public Health Andrija Štampar, University of Zagreb, School of Medicine, Zagreb, Croatia; 2 Department of Radiology, Special Hospital Agram, Zagreb, Croatia; 3 Department of Radiology, University Hospital Center Zagreb, Zagreb, Croatia; 4 Department of Radiology, General Hospital Varaždin, Varaždin, Croatia; 5 Division of Clinical Immunology and Rheumatology, Department of Internal Medicine, University Hospital Centre Zagreb and University of Zagreb School of Medicine, Zagreb, Croatia; 6 Central Medical Library, University of Zagreb, School of Medicine, Zagreb, Croatia; 7 Department of Diagnostic and Interventional Radiology, University Hospital Dubrava, Zagreb, Croatia

## Abstract

**Aim:**

To assess the variation in the waiting time for diagnostic imaging (DI) services among Croatian public hospitals and the utilization of computed tomography (CT) and magnetic resonance imaging (MRI) scanners.

**Methods:**

We analyzed aggregated data from public hospitals. Counties were classified according to economic strength, and utilization was expressed as the average number of exams per machine. We compared the waiting times for 2018 and utilization for 2015 according to hospital category (high and low level) and economic strength by county.

**Results:**

The waiting time was longer for MRI compared with CT, 268 vs 77.61 days. Overall CT waiting time was in the unfavorable European Health Consumer Index category. High-level hospitals had longer waiting time for MRI and CT. The waiting time positively correlated with economic strength for MRI (*P* = 0.019), but not for CT. In low-level hospitals, MRI utilization ranged from 104 to 6032, whereas CT utilization ranged from 48 to 17 852. In high-level hospitals, MRI utilization ranged from 3846 to 11 026, while CT utilization ranged from 503 to 17 234. CT (*P* = 0.041) and MRI (*P* = 0.031) utilization in high-level hospitals was significantly higher than in low-level hospitals.

**Conclusion:**

The waiting times for CT and MRI were exceptionally long regardless of the hospital category, with highly varying utilization. Croatia performed more exams per scanner compared with other EU countries, but not significantly so. High-level hospitals' utilization was significantly higher than that of low-level hospitals, and CT utilization was significantly higher than EU average, while the difference for MRI utilization was not significant.

Radiology has a pivotal position within the health care system owing to the indispensable role of diagnostic imaging (DI) procedures, in particular computed tomography (CT) and magnetic resonance imaging (MRI) in current diagnostic and follow-up algorithms. The demand for DI is continuously growing, expectedly prolonging the waiting time. Waiting time is a topic of high interest when evaluating health system performance, therefore health services need to utilize CT and MRI scanners to the fullest extent. The assessment and minimization of waiting time is of utmost importance not only from a medical but also from a social and political perspective. Long waiting times for medical procedures represent a major obstacle toward achieving optimal (or even standard) levels of care in public health care systems (1-3). Waiting for diagnostic tests accounts for the majority of lengthy waits in the public health care system, leading to lower patients’ satisfaction. Besides affecting patients’ physical and mental health, waiting time also has economic implications for the individual patient and society (4). Achieving a more efficient and high-performing health care system represents a primary concern for most patients and a major aim for health authorities. Implementation of good radiological practice can be an efficient strategy to reduce inefficient and unnecessary spending in health care. Although Croatian counties differ in available radiological and economic resources, the association of these factors with waiting time for DI and characteristics of waiting time have not been studied thus far.

This article aims to assess the variation in waiting time for CT and MRI services across different hospital categories, and the level of utilization and its association with counties' economic strength in Croatia.

## Material and methods

Data from this cross-sectional study were analyzed in June 2019. The most recent administrative data regarding waiting time and utilization of CT and MRI scanners, aggregated per public hospitals in two databases for 2015/2016 and 2018, were obtained from the Ministry of Health. Utilization data were provided for 2015 and expressed as annual mean or median number of exams per machine per hospital. The database for 2018 included the following variables: average waiting time per hospital for any CT and MRI exam, the number of scheduled exams, and the total number of patients waiting for DI services.

### Waiting time

To estimate the period prevalence of patients waiting for MRI and CT services for January 2018, we divided the number of patients by county population and expressed it as proportion (5). The waiting time for MRI scanners was available for all public hospitals. CT waiting time was not available for two community health centers: Zagreb Center Medveščak and Health Center of Split Dalmatia County – Hvar. However, this did not influence the results since these centers were not included in the study. Additionally, we calculated high-to-low ratio as a ratio between the longest and the shortest waiting time.

According to Euro Health Consumer Index (EHCI) 2018, the waiting time for CT scans is defined as the period between the physician’s decision that the scan is needed and the time point when the patient actually undergoes the examination without having to go to a private clinic and further categorized into: less than 7 days (good = up to 1), from 7 to 21 days (intermediate = up to 2), and more than 21 days (bad = 3) (6).

The majority of health care facilities in Croatia providing radiological scanning are publicly owned. The ownership is decentralized, and the central government owns the vast majority of tertiary care hospitals, ie, university hospital centers, university hospitals, and special university hospital/clinics, while counties own secondary care general, county, and specialized hospitals. For the purpose of analysis we categorized hospitals according to organization hierarchy into high-level (university hospital centers, university hospitals, clinical hospitals, and special university hospital/clinics) and low-level hospitals (general, county, and specialized hospitals). We analyzed the association between the waiting time for CT and MRI and counties' economic strength.

### Counties' economic strength

Economic strength was expressed by means of the Croatian Chamber of Economy (CCE) index for 2017, a composite measure that represents the sum of the weighted ranks of GDP per capita, entrepreneurs’ total revenues per employee, entrepreneurs’ revenue on foreign markets per employee, entrepreneurs’ net profit per employee, average net salary, unemployment rate, and projections of population growth 2013-2030 (7). For the purpose of analysis, we presented data descriptively as median with interquartile range (IQR) and merged quartiles Q1 and Q2 into a higher economic strength category and Q3 and Q4 into a lower economic strength category.

### Utilization

Utilization was expressed as the mean or median number of exams per machine per year in hospitals with more than one scanner for 2015. The mean number was estimated as the absolute number of scans divided by the number of machines for a given hospital. We compared MRI and CT utilization between low and high-level hospitals and between counties, and with the estimated average utilization per country obtained from the Eurostat database (EU-28) for 2015. Data for Belgium, Ireland, Greece, Italy, Sweden, and United Kingdom were missing (8).

### Statistical analysis

The results are presented numerically and graphically. Maximum variations in waiting time were calculated for the analyzed year as the ratio between the longest and shortest waiting time. The Shapiro-Wilk test was used to assess the normality of distribution. Numerical data are presented as mean and standard deviation (SD) or as median and IQR. Continuous data were analyzed with *t* test or Mann-Whitney U test. We used Pearson correlation to measure the strength and direction of the association between CT and MRI waiting time and the level of economic strength. The level of statistical significance was set at 0.05. The analyses were performed with IBM SPSS, version 27 (IBM, Armonk, NY, USA).

## Results

The cross-county prevalence of patients waiting for DI services ranged from 0.04% to 0.48% for CT and from 0.02% to 1.88% for MRI. Overall, there were 10 hospitals in the high-level category and 26 in the low-level category, with 58 CT and 26 MRI scanners (Table 1). The waiting time for CT fulfilled the criteria for normal distribution as opposed to MRI (*P* value, 0.096 vs 0.034, respectively). The median waiting time for MRI was 288.50 days (IQR 160.20-373.52), with the mean of 268.00 days (SD = 124.59). The waiting time for MRI ranged from 90 to 419 days (high-to-low ratio 4.65), while the mean waiting time for CT was 77.61 days (SD = 40.68) and ranged from 17 to 154 days (high-to-low ratio of 9.05). Patients waited significantly longer for a MRI scan (*P* < 0.001). There was no significant difference in the waiting time for CT and MRI according to hospital category, although high-level hospitals had longer waiting time (Table 2).

**Table 1:** Croatian Chamber of Economy **(**CCE) indexes, hospitals, hospital category levels, and scanners per counties (2018)*^†^

Q	CCE index (2017)	County	Hospital	Hospital category level	CT	MRI
Q1	67.70	Virovitica-Podravina	General Hospital Virovitica	low	2	0
	67.90	Požega-Slavonia	General Hospital Požega	low	1	1
			General County Hospital Pakrac	low	1	1
	68.00	Bjelovar-Bilogora	General Hospital Bjelovar	low	1	1
	71.60	Brod-Posavina	General Hospital Slavonski Brod	low	1	1
			General Hospital Nova Gradiška	low	1	0
	76.00	Lika-Senj	General Hospital Gospić	low	1	1
	77.30	Vukovar-Srijem	General County Hospital Vukovar	low	1	1
			General County Hospital Vinkovci	low	1	0
Q2	77.60	Šibenik-Knin	General Hospital Šibenik	low	1	0
			General Hospital Knin	low	1	0
	79.70	Sisak-Moslavina	General Hospital Sisak	low	1	0
	80.80	Split-Dalmatia	University Hospital Centre Split	high	4	2
	80.90	Osijek-Baranja	University Hospital Centre Osijek	high	2	2
			General County Hospital Našice	low	1	0
	85.80	Karlovac	General Hospital Karlovac	low	1	0
			General Hospital Ogulin	low	1	0
Q3	87.10	Krapina-Zagorje	General Hospital Zabok	low	2	0
			Specialized Hospital Krapinske Toplice	low	1	0
	90.70	Koprivnica-Križevci	General Hospital Koprivnica	low	1	0
	91.00	Međimurje	County Hospital Čakovec	low	1	0
	91.30	Zadar	General Hospital Zadar	low	2	1
	92.10	Dubrovnik-Neretva	General Hospital Dubrovnik	low	2	1
Q4	95.20	Zagreb^‡^	-	-	-	-
	99.60	Varaždin	General Hospital Varaždin	low	2	0
	105.50	Primorje-Gorski Kotar	University Hospital Centre Rijeka	high	3	2
			Specialized hospital Thalassotherapia Opatija	low	1	1
	127.30	Istria	General Hospital Pula	low	2	0
	147.60	City of Zagreb	University Hospital Centre Zagreb	high	6	2
			University Hospital Centre “*Sestre Milosrdnice*” Clinical Hospital for Traumatology University Hospital for Tumors	high	4	4
			Clinical Hospital Dubrava	high	3	2
			Clinical Hospital Merkur	high	2	1
			Clinical Hospital “*Sveti Duh*”	high	2	1
			Specialized University Children’s Hospital Zagreb	high	1	1
			University Hospital for Infectious Diseases “*Dr. Fran Mihaljević*”	high	1	0
		N = 21	N = 36		N = 58	N = 26

*Q – quartile; CT – computed tomography; MRI – magnetic resonance imaging.

†CCE median = 85.80.

‡Zagreb County does not have hospitals.

**Table 2:** Computed tomography (CT) and magnetic resonance imaging (MRI) waiting time in days according to hospital category

		Low-level hospitals	High-level hospitals	*P*
**CT**	hospitals	N = 26	N = 10	0.318
	mean (SD*)	72.84 (37.28)	89.19 (48.43)	
**MRI**	hospitals	N = 9	N = 9	0.279
	mean (SD)	235.33 (131.83)	300.66 (114.91)	

*SD – standard deviation.

There was an increasing trend of waiting time for MRI imaging according to economic strength of the county. The MRI waiting time positively correlated with CCE index categories (*P* = 0.016), as opposed to CT waiting time (*P* = 0.211). High-to-low ratio was more noticeable for CT waiting time (Table 3). Waiting time was longer for DI services in counties with higher economic strength, significantly for MRI (*P* = 0.019), but not for CT (*P* = 0.202) (Table 4).

**Table 3:** Computed tomography (CT) and magnetic resonance imaging (MRI) waiting time according to the Croatian Chamber of Economy (CCE) index quartiles

Scanner	CCE index (quartiles)	N*	X̅ (standard deviation)/d	Shortest wait (days)	Longest wait (days)	High-to low ratio
MRI	Q1 Q2	6	180.15 (102.651)	90	326	3.62
		2	235.65 (201.738)	93	378	4.06
	Q3 Q4	2	309.30 (153.584)	201	418	2.07
		8	331.64 (94.633)	147	419	2.85
CT	Q1 Q2	9	65.98 (36.984)	20	140	7.00
		8	74.69 (50.985)	17	150	8.82
	Q3 Q4	6	78.70 (32.810)	36	117	3.25
		11	89.50 (42.315)	22	154	7.00

*N – number of hospitals.

**Table 4:** Mean waiting time in days for computed tomography (CT) and magnetic resonance imaging (MRI) according to counties economic strength

	Lower economic strength	Higher economic strength	*P*
MRI	194.03, 95% CI* [95.10, 292.95]	327.17, 95% CI [256.81, 397.53]	0.019
CT	69.02, 95% CI [47.9, 90.14]	87.98, 95% CI [65.39, 110.57]	0.202

*CI – confidence interval.

Utilization intensity considerably varied. In low-level hospitals, MRI utilization ranged from 104 to 6032, while in high-level hospitals it ranged from 3846 to 11 026. CT utilization ranged from 48 to 17 852 in low-level hospitals and from 503 to 17 234 in high-level hospitals.

On average, Croatia had higher utilization of MRI and CT compared with other EU countries, but this difference was not significant. High-level hospitals utilized significantly more CT and MRI scanners compared with low-level hospitals. They also utilized significantly more CT scanners compared with the EU average. However, when high and low-level hospitals were analyzed together, there was no significant difference in the utilization of MRI and CT between the EU and Croatia (*P* = 0.489 and *P* = 0.826, respectively). Economic strength was not significantly associated with MRI and CT utilization (Table 5 and Table 6).

**Table 5:** Utilization of computed tomography (CT) and magnetic resonance imaging (MRI) according to hospital categories for 2015

Scanners	Low-level hospitals	High-level hospitals	Croatia^†^	EU^‡^
MRI				
mean (SD)	3783.77 (1626.51)	5557.77 (2167.84)	4759.47 (2099.34)	4472.14 (2365.43)
median (IQR)		4599.50 (4146-5967.50)	4297.50 (3827.50-5475)	
CT				
mean (SD)	6068.80 (4541.71)	9984.78 (5135.70)	7187.65 (4976.31)	6062.50 (3085.00)
median (IQR)	5300 (2667-7595)	.	6354 (3003-10 885.75)	

*SD – standard deviation; IQR – interquartile range.

†data obtained from the Croatian Ministry of Health for 2015.

‡Eurostat data 2015.

**Table 6:** Utilization differences according to computed tomography (CT) and magnetic resonance imaging (MRI)

	*P* value	
	CT	MRI
Croatia mean vs EU mean	0.489	0.826
High-level vs low-level hospitals	0.041	0.031
High-level hospitals vs EU mean	0.045	0.254
Low-level hospital vs EU mean	0.996	0.435
High-level vs low-level counties	0.109	0.184

## Discussion

This is the first comprehensive quantitative analysis of waiting time and utilization of radiological services in Croatia. Diagnostic radiology is the fastest growing sector in terms of medical costs, and its high-value medical equipment has a central role in clinical decision-making (9,10). Waiting time represents the top health service concern among the general public, and is the subject of regular public and political debate. The burden of patients waiting for diagnostic imaging service in our study was similar to that in other European countries and ranged from 0.04% to 0.48% for CT and from 0.02% to 1.88% for MRI (11). Long waiting times for scheduled health services in public hospitals can be viewed as a “fact of life” because the demand for subsidized health care by far exceeds the supply. Our results indicate significantly longer MRI waiting time compared with CT and exceptionally high magnitudes of both waiting times. This raises the question of sustainability, ie, whether a high (or even sufficient) standard of health care can be provided in the long run. Waiting time analysis also represents an important component of the assessment of health care system performance. Accordingly, some governments defined waiting time standards for key diagnostic and/or follow-up tests and investigations, and even established waiting time warranty at the national level. Croatia, however, has not yet developed such standards. Comparison of health care standards among countries may be demanding and limited, but it is noteworthy that in France a waiting time of 40 days is considered unacceptable or even alarming (12). The Netherlands is also setting an example as since 2010 MRI waiting time has not exceeded three weeks (13).

**Figure 1:**
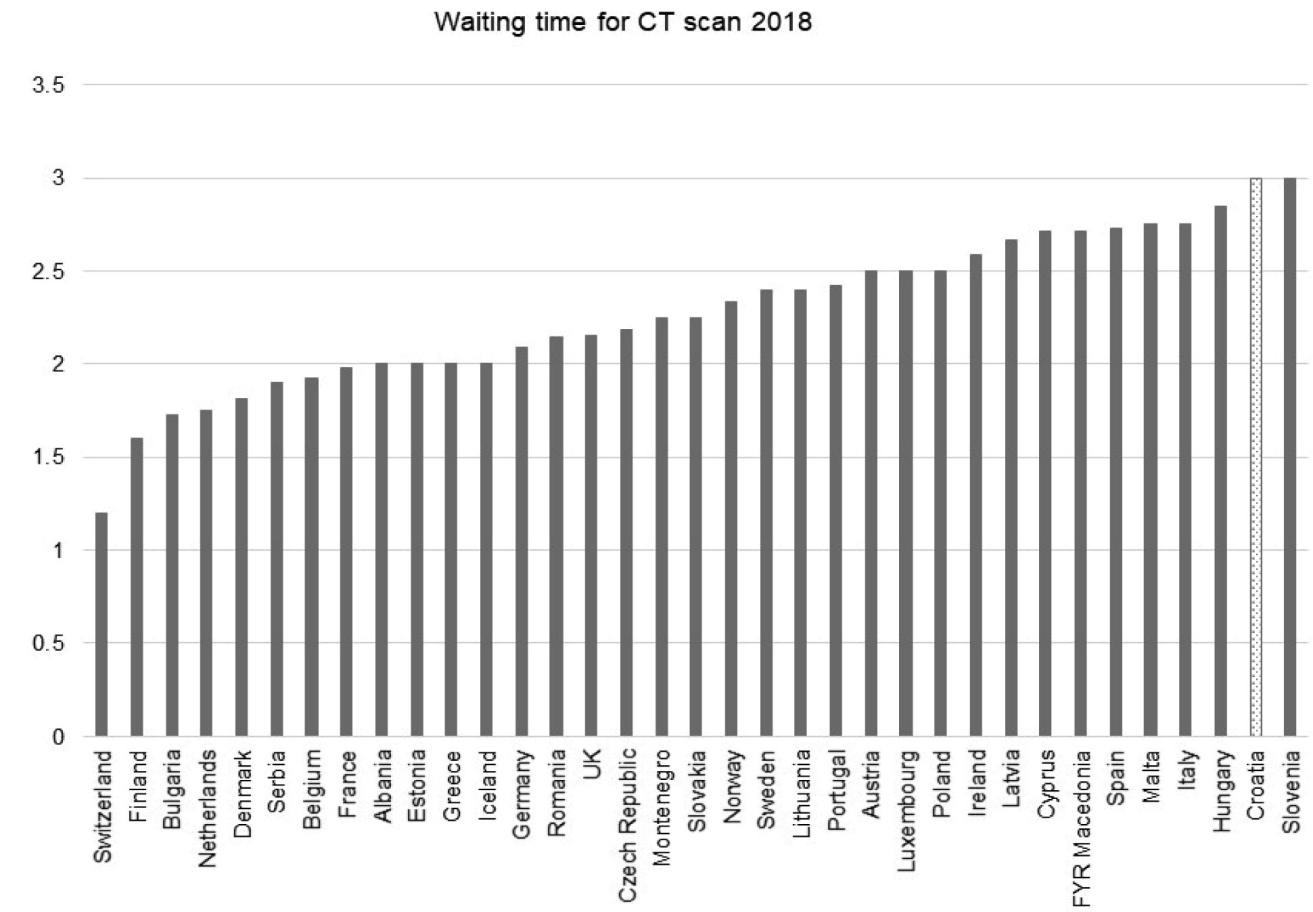
Waiting times for computed tomography (European Health Consumer Index 2018). x-axis: country; y-axis: European Health Consumer Index (categorization of waiting time): 3 = bad (waiting time >21 days), up to 2 = intermediate (waiting time 7-21 days), up to 1 = good (waiting time <7 days).

EHCI provides data on indicators of health system performance and waiting times for CT diagnostic procedures in European countries. Croatia is at the bottom of the list since 93.5% of hospitals have waiting times longer than 21 days (Figure 1). There is lack of supporting data, but such “provider delay” may likely be associated with poor patients’ outcome. A potential remedy for the current practice could be to stratify patients according to indications (eg, oncological vs non-oncological, diagnostic vs follow-up) and anatomical regions (eg, head/neck, thorax/abdomen, musculoskeletal etc). Prioritization is a key issue, and implementation of evidence-based protocols could reduce harm from long waiting time.

The length of MRI and CT waiting lists is affected by numerous factors, the most important being limited national health care budget and availability of scanners per 100 000 population. There are no general guidelines regarding the ideal number of CT or MRI scanners per population. Still, if compared with the EU average per 100 000 population as a reference capacity indicator, Croatia has a lower capacity, with 1.8 vs 2.2 for CT and 1.1 vs 1.4 for MRI scanners (6). The low supply of MRI scanners is especially undesirable, since MRI can be used instead of ionizing radiation in a variety of clinical circumstances (14). Moreover, overutilization of CT scanners exposes patients to unnecessary radiation doses and increases the medical exposure dose in the population. Unfortunately, recommendations that offer guidance as to when CT is indicated are poorly implemented in clinical practice. Furthermore, Croatia is inhomogeneous regarding the accessibility and quality of health care (15). Such inhomogeneity is illustrated by the high-to-low ratio of 4.65 for MRI and 9.05 for CT in our study. This is not unexpected since hospitals differ in size, the number of CT and MRI scanners, radiology personnel, and practice pattern. In 2018, there was an unequal distribution of DI services between the public and private sector. Regarding the CT service, 60 machines were publicly owned and 17 were privately owned, while as many as 25 of 51 MRI scanners were privately owned. Private providers in most instances offer out-of-pocket services but only occasionally they had outsourced arrangements with the public sector. Although it can be assumed that the radiology service offered by private health care providers could efficiently reduce waiting lists, cost-utility analyses conducted in other countries and medical fields are not available (16,17).

### Waiting time for computed tomography and magnetic resonance imaging according to hospital categories and economic strength

The association between CT and MRI waiting time and hospital categorization was positive but non-significant. High-level centers supplied insufficient level of diagnostic services compared with patients’ demand, thus increasing the waiting times. A similar trend has already been observed in previous studies (18-21). Such disequilibrium between supply and demand can be explained by the quality of medical facilities, quantity and quality of medical equipment, capacity of human resources, and patient flow. Patients may prefer large centers because of better equipment and higher level of expertise in subspecialty areas. Although ownership of health care facilities has been divided between the central government and counties, the use of services in high-level medical institutions, especially university hospital centers, is not restricted by health care providers. Such unlimited access is most likely associated with overutilization of imaging services for both diagnostic and therapeutic purposes, however evidence supporting this claim is still lacking.

Economic strength was positively associated with waiting time. High-economic strength was significantly associated with longer MRI waiting time, however for CT the association was positive but not significant. Although low income is associated with poorer health, economically privileged counties experienced increased demand. Such a trend has been present for a longer period. In the past, strategies for dealing with long waiting lists primarily involved referring patients to hospitals with shorter waiting lists. The final outcomes were not analyzed, but it is unlikely that all patients could have benefited from this. Some of them are unable to travel due to illness, distance to the hospital, or high travel expenses, the latter being perceived as a very serious problem in Croatia (4). As previously described, the utilization of services is inversely associated with the distance of the patient's place of residence from the hospital, which is called “distance decay” (22). The role of “distance decay” does not contradict with overutilization of high-level hospitals: the majority of the population lives in urban or suburban areas in the vicinity of university hospital centers.

The length of waiting lists is affected by many additional factors. Increased number of radiological exams could be explained by high population density, differences in disease prevalence, and health needs of the population. The shortage of equipment and radiological workforce, repeated examinations, examinations performed although unlikely to affect patient management, and premature or incorrect examinations were previously described (15).

Our results confirmed cross-county disparity in radiological workforce but its true effect on imaging service is difficult to estimate. For instance, although the overall number of radiologists is available, the true number of those qualified for reporting of CT/MRI imaging is not available. Of note, teleradiology services are occasionally used to fill the capacity gap but their potential role in reducing waiting time could not be analyzed since there is no mandatory report of its usage.

### Computed tomography and magnetic resonance imaging utilization

A previous study reported underutilized capacities in suburban and rural areas, but the source of evidence was characterized as anecdotal (23). Our findings also confirmed large variability in scanner utilization, which was slightly less pronounced in high-level hospitals. High-to-low ratio for CT in low-level hospitals was exceptionally high compared with high-level hospitals. A similar difference was observed for MRI. In other words, these findings confirmed an urgent need for a more efficient DI service, especially in low-level hospitals. Each country has a unique structure of health care system and evaluation criteria, and there is a lack of widely accepted performance standards for CT and MRI utilization. Despite the provision of comparable DI services, the question of reasonable utilization level cannot be easily answered. Still, for the purpose of comparison, average EU level of MRI and CT utilization can be estimated from available Eurostat data at 4472.14 and 6062.5 exams per scanner per year.

Utilization of CT scanners varies also across Europe. Hungary, France, and Luxembourg had the most intensive use (more than 11 000 scans per machine), while Bulgaria, Finland, and Romania had the least intensive use, each with an average of only 2200 scans per CT scanner or even fewer (7).

In regard to MRI utilization, it was highest in Hungary (10 600 scans per machine), followed by France (8400 scans per machine). Cyprus had the least intensive use, as each MRI machine was used on average 348 times. It was followed by Bulgaria (1068 scans per unit) (7). Utilization of MRI and CT in Croatia was non-significantly higher compared with the EU average. High-level hospitals utilized CT and MRI scanners significantly more compared with low-level hospitals. Furthermore, they utilized CT scanners significantly more compared with the EU average. Economic strength was not significantly associated with MRI and CT utilization.

In general, underutilization in low-level hospitals indicates that there is no need to buy more scanners. Underutilization can be influenced by both supply and demand. It is unquestionably associated with the shortage of radiographers and radiologists, but there is a lack of disaggregated hospital data to quantify such association. Small counties are mostly affected by uneven geographical distribution of radiologists, while large medical centers face increased demand for diagnostic services. To provide radiological services, many small centers regularly employ consultants, who work at multiple sites and narrow the capacity gap. The number of radiological personnel is often reported as a total number without considering radiologists' subspecialty training in MRI or CT. There could be a limited number of radiologists to interpret the results. Daily practice of older radiologists often includes performing other imaging modalities, such as x-rays and ultrasounds. Supply issues could include restrictions in working time and an increasing preference for women to work day shifts. Regarding demanding constrains, some hospitals, such as special pediatric clinics or hospitals for infectious diseases, may be unable to achieve average (or sufficient) levels of utilization because of low patients’ number.

### Limitations

The major strength of this study is that waiting time and utilization level for MRI and CT scans have been analyzed in-depth at a cross-county level and compared with other countries according to previously published criteria.

The EHCI definition of waiting time used in this study allowed cross-country comparison. The time between referral and examination is called the pre-examination waiting time. We are aware that total radiology waiting time should be included in the finalized radiology report, but these data were not available.

In addition, administrative data were collected per medical institution per one time period but additional data were not provided. We were also not able to analyze annual variations. Public and private hospital databases and mortality database are not linked, so there is no possibility to cross-check patients' data. Registration at several lists, the use of private services, patient’s death, or omitting to cancel the reservation could have lead to the waiting time overestimation. Since reported data are not patient-based, correlations between waiting time and economic power are also provided on an aggregated level.

This article analyzed the utilization of MRI and CT scanners focusing on just one indicator, the annual number of exams per scanner, while the number of operating hours per scanner was not available. Such aggregated data cannot sufficiently explain the variability or be used to estimate the efficiency of scanners, taking into account operating hours. Nevertheless, they can be indicative of potential workload.

Still, the length of exams also varies depending on the type of examination procedure. In other words, MRI exams are generally more complex and time-consuming compared with CT exams. Another important issue is that waiting time is most likely increased by unnecessary and repeated examinations. The exact impact of such misuse cannot be estimated given that it has not been investigated to date.

### Conclusion

The waiting times for CT and MRI in Croatia are exceptionally long regardless of the hospital category, with high variations in the utilization level. On average, Croatia performs more exams per scanner compared with the EU countries. High-level hospitals utilized scanners significantly more than low-level hospitals. With regard to CT scanning, high-level hospitals performed significantly above the EU average, but still the waiting time remained the longest among European countries. Insufficient capacity to meet the patient demand could be partly attributed to regional disparities in human and technical resources. Rather than the lack of capacity, the major challenge seems to be ineffective capacity planning that does not take into account regional and seasonal demands and variations. Furthermore, the potential impact of inappropriate indications for DI services on waiting time should be addressed in the future, leading to responsible utilization of radiological resources according to appropriate clinical criteria.
